# Comparison of repeat penetrating keratoplasty, DSAEK and DMEK for the management of endothelial failure of previous PK

**DOI:** 10.1038/s41433-023-02561-5

**Published:** 2023-06-02

**Authors:** Harry W. Roberts, Laura de Benito-Llopis

**Affiliations:** 1 Corneal and External Diseases Unit, Moorfields Eye Hospital NHS Foundation Trust, London, UK; 2 West of England Eye Unit, Royal Devon University Healthcare NHS Foundation Trust, Exeter, UK; 3 University of Exeter Medical School, Exeter, UK

**Keywords:** Corneal diseases, Prognosis

## Abstract

**Purpose:**

To compare the clinical outcomes of repeat PK, DSAEK-on-PK or DMEK-on-PK for the management of endothelial failure of previous penetrating keratoplasty.

**Design:**

Retrospective, interventional consecutive case series.

**Participants:**

104 consecutive eyes of 100 patients requiring a second keratoplasty for endothelial failure of their primary penetrating keratoplasty performed between September 2016 and December 2020.

**Intervention:**

Repeat keratoplasty.

**Main outcome measures:**

Survival and visual acuity at 12 and 24 months, rebubbling rate and complications.

**Results:**

Repeat PK was performed in 61/104 eyes (58.7%), DSAEK-on-PK was performed in 21/104 eyes (20.2%) and DMEK-on-PK was performed in 22/104 eyes (21.2%). Failure rates in the first 12 and 24 months were 6.6% and 20.6% for repeat PKs compared to 19% and 30.6% for DSAEK and 36.4% and 41.3% for DMEK. For those grafts surviving 12 months, the chances of surviving to 24 months were greatest for DMEK-on-PK at 92% vs 85% each for redo PK and DSAEK-on-PK. Visual acuity at one year was logMAR 0.53 ± 0.51 in the redo PK group, 0.25 ± 0.17 for DSAEK-on-PK and 0.30 ± 0.38 for DMEK-on-PK. 24-month outcomes were 0.34 ± 0.28, 0.08 ± 0.16, and 0.36 ± 0.36 respectively.

**Conclusions:**

DMEK-on-PK has a greater failure rate in the first 12 months than DSAEK-on-PK which has a greater failure rate than redo PK. However, the 2-year survival rates in our series for those already surviving 12 months were greatest for DMEK-on-PK. There was no significant difference in visual acuity at 12 or 24 months. Careful patient selection is needed by experienced surgeons to determine which procedure to offer to patients.

## Introduction

Despite the rise of lamellar keratoplasty techniques in the last two decades, penetrating keratoplasty (PK) is still commonly performed and remains the procedure of choice for selected indications by many surgeons [1]. Depending on the indication for surgery, a primary PK may have a median survival time of between 15 and 20 years [2]. Despite this apparent longevity, many PK recipients may ‘outlive’ their graft and require further keratoplasty. Repeat transplant is therefore one of the most common indications for transplant and in the United States is the second most common indication for PK and the fourth most common indication for endothelial keratoplasty (EK) [1]. In the United Kingdom, regrafts represent 21.1% of all corneal transplants between 1999-2016 and 19.1% in the West of Scotland between 2001–2010 [3, 4].

As endothelial failure (whether primary failure, late decompensation, or irreversible rejection) of the PK represents the majority of indications for regraft, then surgical options for management of these cases include repeat PK, Descemet stripping (automated) endothelial keratoplasty (DSAEK/DSEK) or Descemet membrane endothelial keratoplasty (DMEK) [2]. While many case series of outcomes for a single technique have been published, comparative studies have been relatively few and either compare DSAEK-on-PK vs. redo PK or DMEK-on-PK vs. DSAEK-on-PK [5–8]. To date, we are not aware of any study comparing clinical outcomes of all three treatment modalities in one series.

The aim of this study was to compare the 12- and 24-month outcomes of redo PK vs DSAEK-on-PK vs DMEK-on-PK for endothelial failure of first PK among multiple surgeons at Moorfields Eye Hospital, London, UK.

## Methods

This study was approved as a clinical audit report by the Clinical Audit Committee at Moorfields Eye Hospital, London (CA22/CED/920) and was performed in accordance with the tenets of the Declaration of Helsinki. Informed consent to collect data for audit purposes was obtained before surgery as part of routine clinical practice. At Moorfields, all patients undergoing corneal graft surgery attend a follow-up appointment at 12 and 24 months after surgery at the Graft Outcome Clinic, where the status of the graft is recorded, in addition to visual acuity, complications and other clinical data. This is then introduced into the Moorfields Corneal Graft Database.

This study was an institutional retrospective cohort study performed at Moorfields Eye Hospital, London, UK, with data extracted from the Moorfields Corneal Graft Database. We also reviewed case notes and electronic records from the Moorfields Electronic Healthcare Record System (OpenEyes, Apperta Foundation CIC, Sunderland, UK) to obtain information not available in the database.

We included all eyes that underwent PK, DSAEK or DMEK between September 2016 and December 2020 with a past history of only one PK in the operated eye. Indication for surgery was endothelial failure of the primary PK from any cause (primary failure, immunological rejection, late endothelial failure). Eyes with previous lamellar graft surgery or more than one PK in the operated eye were not included i.e. this was the second keratoplasty for each eye in the study. Only cases with minimum 1-year follow-up and complete data available were included. We only included the initial graft performed during the audit period for each eye. As this is a retrospective database study, decision to proceed with redo PK, DSAEK-on-PK or DMEK-on-PK was an individual decision made by each consultant surgeon based on factors such as visual potential, relative risks, anterior lamellar profile and scarring among others.

### Statistical analysis

All data collected in the study were entered into an electronic database via Microsoft Excel 2007 (Microsoft Corp., Redmond, WA), and statistical analyses were performed using SPSS Statistics Version 16 (IBM, Armonk, New York, USA). Descriptive statistics was used to calculate averages and standard deviation of the performances in each list. Differences in baseline characteristics were tested with Chi-squared. For normally distributed continuous data, 1-way analysis of variance was used to determine significant differences between the means of the three groups. Post hoc Tukey test was applied to perform comparisons between groups. *P* values less than 0.05 were considered statistically significant. A Kaplan-Meier survival analysis using log-rank test was conducted to compare the survival probabilities of the DMEK, DSAEK, and PK groups. Binary logistic regression analysis was then performed for transplant survival at one year using independent variables from the baseline demographics that reached a significant level of less than 0.05 in univariate analysis. The normit link function was chosen as it produced the best goodness of fit results. Variables that reached a significant level of less than 0.05 in multivariate analysis were considered significant.

## Results

Overall, 104 eyes of 100 patients who underwent repeat PK, DSAEK or DMEK during the study period were included in the analysis. Demographics, indications for surgery and main baseline clinical data of the study population are described in Table 1.

**Table 1:** Baseline demographic and clinical characteristics of patients undergoing regraft and separated according to the technique (redo PK vs DSAEK-on-PK vs DMEK-on-PK).

	PK *N* = 61	DSAEK *n* = 21	DMEK *n* = 22	Total *n* = 104	*P*=
Age at surgery (Mean ± SD)	55.4 ± 19.5	60.3 ± 12.7	56.6 ± 14.3	56.6 ± 17.5	0.34
Laterality (% Right)	52.8% (*n*=32)	38.8% (*n*=8)	63.6% (*n*=14)	51.7% (*n*=54)	0.24
Gender (%male)	60.7% (*n*=37)	71.4% (*n*=15)	63.6% (*n*=14)	63.46% (*n*=66)	0.67
Indication for original PK					0.34
Keratoconus	19/61 (31.1%)	7/21 (33.3%)	13/22 (59.1%)	39/104 (37.5%)	
BK	11/61 (18.0%)	3/21 (14.3%)	1/22 (4.5%)	15/104 (14.4%)	
FED	0/61 (0%)	1/21 (4.8%)	2/22 (9.1%)	3/104 (2.9%)	
Other dystrophies	3/61 (4.9%)	1/21 (4.8%)	2/22 (9.1%)	6/104 (5.8%)	
Bacterial Infection	1/61 (1.6%)	1/21(4.8%)	0/22 (0%)	2/104 (1.9%)	
Fungal Infection	3/61 (4.9%)	0/21 (0%)	0/22 (0%)	3/104 (2.9%)	
Protozoal Infection	2/61 (3.3%)	1/21(4.8%)	0/22 (0%)	3/104 (2.9%)	
Viral Infection	2/61 (3.3%)	0/21 (0%)	1/22 (4.5%)	3/104 (2.9%)	
Mechanical Injury	2/61 (3.3%)	0/21 (0%)	1/22 (4.5%)	3/104 (2.9%)	
Other/Unknown	18/61 (29.5%)	7/21 (33.3%)	2/22 (9.1%)	27/104 (26.0%)	
Graft Diameter (mm)	8.02 ± 0.64	7.77 ± 0.35	7.52 ± 0.39	7.86 ± 0.62	0.04
Visually significant comorbidity					<0.001
Yes	44/61 (72.0%)	10/21 (47.6%)	5/22 (22.7%)	59/104 (56.7%)	
No	17/61 (28.0%)	11/21 (52.4%)	17/22 (77.3%)	45/104 (43.3%)	
Grade of Surgeon					0.91
Consultant	35/61 (57.4%)	13/21 (61.9%)	11/22 (50.0%)	59/104 (56.7%)	
Fellow	22/61 (36.1%)	6/21 (28.6%)	9/22 (40.9%)	37/104 (35.6%)	
Other	4/61 (6.6%)	2/21 (9.5%)	2/22 (9.1%)	8/104 (7.7%)	
Secondary procedures at time of surgery					0.54
None	41/61 (67.2%)	13/21 (76.2%)	17/22 77.3%	74/104 (71.2%)	
Phacoemulsification and IOL implantation	6/61 (9.8%)	3/21 (14.3%)	1/22 (4.5%)	10/104 (9.6%)	
IOL exchange	5/61 (8.2)%	0%	0%	5/104 (4.8%)	
Vitrectomy	5/61 (8.2%)	0%	1/22 (4.5%)	6/104 (5.8%)	
Glaucoma Surgery	2/61 (3.3%)	0%	0/22 (0%)	2/104 (1.9%)	
Others	4/61 (6.6%)	3/21 14.3%	2/22 (9.1%)	9/104 (8.7%)	

*PK* penetrating keratoplasty, *DSAEK* descemet stripping automated endothelial keratoplasty, *DMEK* descemet membrane endothelial keratoplasty, *BK* bullous keratopathy, *FED* Fuchs endothelial dystrophy, *IOL* intraocular lens.

The visual outcomes are reported in Table 2A. Eyes that developed graft failure (Table 2B) were excluded from analysis of visual outcome but are included for the rest of the analysis. There was a statistically significant difference at baseline in all eyes having repeat PK having worse pre-operative best-corrected visual acuity (BCVA) than those having DSAEK or DMEK before excluding for visually significant comorbidites. BCVA at 1 and 2 years postop did not show a statistically significant difference between the three groups. Post hoc Tukey tests did not show statistically significant differences between the groups at any point other than baseline.

**Table 2:** A. Best corrected visual acuity (BCVA) pre-operatively and at one and two years post-operatively for redo PK, DSAEK-on-PK, and DMEK-on-PK. B. Kaplan-Meier analysis of graft survival probability (%) of redo PK, DSAEK-on-PK and DMEK-on-PK.

		Follow up duration (Years)		
		Preop	1	2
**A.**				
**BCVA LogMAR**	**All Eyes**			
	PK	1.90 ± 0.97	1.19 ± 0.95	0.94 ± 0.93
	DSAEK	1.24 ± 0.79	0.74 ± 0.78	0.75 ± 1.10
	DMEK	1.17 ± 0.88	0.68 ± 0.78	0.77 ± 0.46
	ANOVA	*F* = 6.49 ***p*** = **0.002**	*F* = 2.47 *p* = 0.092	*F* = 0.17 *p* = 0.846
	Post Hoc Tukey	PK vs DSAEK *p* = 0.02 PK vs DMEK *p* = 0.01 DSAEK vs DMEK *p* = 0.97	PK vs DSAEK *p* = 0.23 PK vs DMEK *p* = 0.15 DSAEK vs DMEK *p* = 0.98	PK vs DSAEK *p* = 0.88 PK vs DMEK *p* = 0.94 DSAEK vs DMEK *p* = 1
	**Eyes with Visually significant comorbidities excluded**			
	PK	1.41 ± 0.89	0.53 ± 0.51	0.34 ± 0.28
	DSAEK	0.72 ± 0.57	0.25 ± 0.17	0.08 ± 0.16
	DMEK	1.23 ± 0.98	0.30 ± 0.38	0.36 ± 0.36
	ANOVA	*F* = 1.79 *p* = 0.179	*F* = 1.35 *p* = 0.278	*F* = 1.84 *p* = 0.18
	Post hoc Tukey	PK vs DSAEK *p* = 0.15 PK vs DMEK *p* = 0.77 DSAEK vs DMEK *p* = 0.46	PK vs DSAEK *p* = 0.32 PK vs DMEK *p* = 0.50 DSAEK vs DMEK *p* = 0.97	PK vs DSAEK *p* = 0.26 PK vs DMEK *p* = 0.99 DSAEK vs DMEK *p* = 0.33

B.						
	Redo-PK		DSAEK		DMEK	
	Number of eyes at risk	Kaplan-Meier survival probability estimate	Number of eyes at risk	Kaplan-Meier survival probability estimate	Number of eyes at risk	Kaplan-Meier survival probability estimate
Year 1	61	93.4%	21	81.0%	22	63.6%
Year 2	40	79.4%	14	69.4%	13	58.7%

*PK* penetrating keratoplasty, *DSAEK* descemet stripping automated endothelial keratoplasty, *DMEK* descemet membrane endothelial keratoplasty.

Bold values denote significant *p* values.

**Fig. 1:**
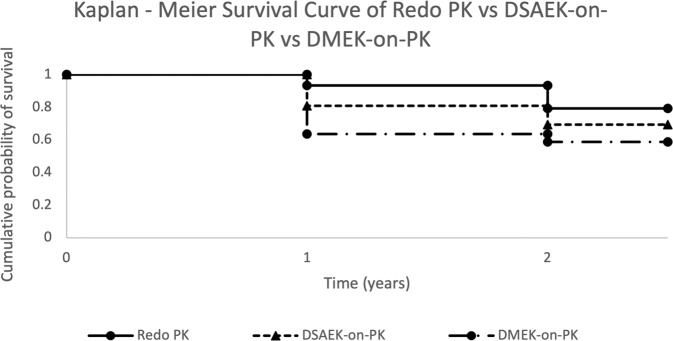
Kaplan–Meier survival curve at 1 and 2 years of redo PK vs DSAEK-on-PK vs DMEK-on-PK. PK penetrating keratoplasty, DSAEK descemet stripping automated endothelial keratoplasty, DMEK descemet membrane endothelial keratoplasty.

Table 2B demonstrates a Kaplan-Meier analysis of graft survival probability. Failure rates in the first 12 and 24 months were 6.6% and 20.6% for repeat PKs compared to 19% and 30.6% for DSAEK and 36.4% and 41.3% for DMEK (Fig. 1). For those grafts surviving 12 months, the chances of surviving to 24 months were greatest for DMEK-on-PK at 92% vs 85% each for redo PK and DSAEK-on-PK.

Postoperative complications are detailed in Table 3. There was no difference between rebubbling rates for DSAEK (19.0%) and DMEK (18.2%). There was one case of endothelial rejection in each group (PK 1.63%, DSAEK 4.8%, DMEK 4.5%).

**Table 3:** Post operative complication rates of redo PK, DSAEK-on-PK and DMEK-on-PK.

	PK	DSAEK	DMEK
Graft detachment requiring rebubbling	0/61 (0%)	4/21 (19.0%)	4/22 (18.2%)
PK wound dehiscence	1/61 (1.6%)	0/21 (0%)	0 (0%)
Endothelial rejection	1/61 (1.6%)	1/21 (4.8%)	1/22 (4.5%)
Infection	0 (0%)	1/21 (4.8%)	0 (0%)

*PK* penetrating keratoplasty, *DSAEK* descemet stripping automated endothelial keratoplasty, *DMEK* descemet membrane endothelial keratoplasty.

Univariate logistic regression returned redo-PK (vs DMEK and DSAEK) and DMEK-on PK (vs PK and DSAEK) as statistically significant risk factors for graft survival at one year (Table 4A). Neither of these was statistically significant on multivariate regression, DMEK-on-PK (OR 0.36, 95% CI 0.08–1.62, *p* = 0.18) and redo PK (OR 2.9, 95% CI 0.49–11.62, *p* = 0.29) (Table 4B).

**Table 4:** Logistic regression of factors predicting failure to survive to 12 months post-operatively.

Parameter	Odds Radio (95% CI)	*P* value
A. Univariate analysis of factors		
Age at surgery (years)	1.02 (0.99–1.06)	0.12
Gender (female vs male)	4 (0.84–18.94)	0.08
DMEK-on-PK (vs others)	0.2 (0.06–0.65)	0.008
DSAEK-on-PK (vs others)	0.92 (0.23–3.63)	0.90
Redo PK (vs others)	3.89 (1.11–13.61)	0.03
Surgery combined with other procedures (vs transplant only)	1.33 (0.34–5.19)	0.68
Grade of surgeon (Consultant vs others)	0.98 (0.31–3.06)	0.97
Presence of visually significant comorbidity	1.39 (0.45–4.34)	0.57
Graft diameter (mm)	1.61 (0.65–4.04)	0.31
**B. Multivariate analysis of factors reaching significance in the univariate analysis**		
DMEK-on-PK (vs others)	0.36 (0.08–1.62)	0.18
Redo PK (vs others)	2.9 (0.49–11.62)	0.29

*PK* penetrating keratoplasty, *DSAEK* descemet stripping automated endothelial keratoplasty, *DMEK* descemet membrane endothelial keratoplasty.

## Discussion

Redo PK, DSAEK-on-PK and DMEK-on-PK are all viable treatment options for endothelial failure of PK although a direct comparison of the three techniques has yet to be published. Studies published to date have either compared DSAEK-on-PK with redo PK, DSAEK-on-PK with DMEK-on-PK or grouped all EK-on PK together. This is the first time to our knowledge that the three treatment options have been directly compared and demonstrates differences in survival rates between each procedure.

The expected longevity of primary PK, DSAEK or DMEK in low-risk recipients can be expected to exceed 5 years in 90% of recipients [2, 9, 10]. However, repeat grafts are known to have a greater failure rate than primary grafts so predicted longevity is of critical importance in offering second keratoplasty to patients [3]. The failure rates we report within the first 12 months of 6.6% (PK), 19% (DSAEK-on-PK) and 36.4% (DMEK-on-PK) are consistent with the literature with reported 12-month failure rates of 2–10% for redo PK, 11.7–36% for DSAEK-on-PK, and 7–37% for DMEK-on-PK [5, 7, 8, 11, 12]. The greater rates of primary failure for the EK arms are likely to be partly as a result that EK-on-PK may be more technically challenging and is also known to have a higher rate of graft detachment than for primary EK with a concurrent increase in rebubbling rates [8]. EK rebubbling rates of 19% for DSAEK-on-PK are consistent with the reported rates in the literature (9-22%) and 18.2% for DMEK-on-PK is lower than the typically higher rate of rebubbling reported (28–56%) [8]. None of the available data collected at baseline was statistically significant in the multivariate logistic regression looking at 12-month transplant survival (Table 4).

Despite the 36.4% of DMEK-on-PK which fail in the first year, the 2-year survival rates in our series for those already surviving 12 months were greatest for DMEK-on-PK at 92% vs 85% each for redo PK and DSAEK-on-PK indicating that after the initial higher rate of failure for EK, the rates of late failure may plateau. There are conflicting reports in the literature on the long-term differences in graft survival for EK-on-PK and redo PK [6, 13]. Ang et al. found a three-year survival rate of 66.8% for redo PK vs 86.4% for EK-on-PK in a study of 113 eyes [13]. In contrast, an Australian graft registry study including 335 redo PKs and 65 EK-on-PK demonstrated a statistically significant inferior graft survival for EK-on-PK [6]. A UK registry study found no difference in survival for EK-on-PK than redo PK for Fuchs endothelial dystrophy and pseudophakic bullous keratopathy [3]. The discrepancy between registry study data and single-centre outcomes may reflect the inclusion of learning curve cases within a registry study and outcomes from surgeons performing low volumes of keratoplasty compared with outcomes from a single centre and variations in thresholds for offering EK-on-PK vs redo PK.

Differences in the patient cohorts and complexity of eyes may also contribute to observed differences. For example, in this study, there were significant differences at baseline which are to be expected given this is a retrospective, non-randomised database study where the selection criteria for the various treatments may vary. Repeat PK was more likely to be performed when there were visually significant co-pathologies and DMEK-on-PK was more likely to have been performed when there were none. This then explains the statistically significant differences in baseline visual acuity before excluding those with visually significant co-pathologies.

Advantages of EK-on-PK include that the previous astigmatic rehabilitation of the primary PK is preserved. This may be beneficial if this eye previously enjoyed a PK with low astigmatism and/or good visual acuity. An EK will offer faster visual rehabilitation than redo PK, and avoids the use of sutures to secure the graft with associated suture-induced astigmatism, complications and the need for suture removal [14]. It also retains rather than resets the existing wound healing across the PK-host junction resulting in a tectonically stronger eye [15]. It is also more common to perform EK under local anaesthesia than PK thus avoiding the potential disadvantages of general anaesthesia [16]. Finally, it may be a better option in the setting of ocular surface disease in which a redo PK may be at high risk of surface failure.

Despite the advantages of EK-on-PK, redo PK has its own advantages. It has the highest rate of 12-month survival in this series and avoids the need to posture post-operatively. Therefore, it is a good option in those who would find it difficult to tolerate post-operative posturing or those who would prefer to minimise the chance of needing further interventions such as rebubbling or regraft for primary failure. Redo PK is the most suitable option for combined endothelial failure with stromal opacity, or where the primary PK had intolerable astigmatism prior to failure. Stromal opacity may, however, be a relative indication for redo PK as it has been demonstrated that stromal opacity secondary to stromal oedema may remodel for 12 months or longer after EK-on-PK [17]. Finally, it may often be the clinically most suitable option in complex anterior segment anatomy in which EK may be technically more challenging.

It is well established that in primary keratoplasty PK has higher risk of rejection than DSAEK which in turn has higher risk of rejection than DMEK [18]. Regrafts do not have such low rates of rejection as primary low-risk grafts, perhaps due to pre-sensitisation of the local mechanisms of immune tolerance and/or vascularisation of the primary PK [19]. While EK-on-PK may have a greater risk of rejection than primary EK, a meta-analysis of 4 studies did demonstrate that EK-on-PK had a lower risk of rejection than redo PK [OR = 0.43 (95% CI: 0.23–0.80, *P* = 0.007)] [20]. Our study had one case of immunological rejection in each group thus lacking sufficient numbers to make meaningful assertions.

We did not find a statistically significant difference in the visual acuity at 1- or 2-years after surgery between the three techniques. As this was not a randomised controlled trial, there would have been heterogeneity between the groups and therefore this study was not designed to test for which treatment offered superior visual outcomes. Our findings were in keeping to the Australian Registry study which did not detect a visual difference between PK-PK and PK-EK [6]. Two single-centre series reported better visual outcomes for DMEK-on-PK than DSAEK-on-PK although in both the DSAEK-on-PK group also had worse baseline visual acuity [5, 7]. Without adequately powered randomised controlled trials it may be difficult to conclude which technique offers the best visual results and decision making will often come down to individual surgeon’s experience and factors such as graft astigmatism, degree of anterior stromal opacity and other risk factors for graft failure/challenging surgery. If we can extrapolate from studies on DMEK, DSAEK and PK in virgin eyes then we would assume that DMEK should be the technique most likely to optimise the visual acuity, however this needs to be balanced against the higher risk of failure within the first 12 months [9]. This can be inferred from the choice of DMEK-on-PK as the most likely procedure to have been performed in those patients without visually significant comorbidity.

In summary, DMEK-on-PK had the highest 12-month failure rates followed by DSAEK-on-PK then redo PK but otherwise, complication profiles were similar. However, the 2-year survival rates for those already surviving 12 months were greatest for DMEK-on-PK compared to redo PK and DSAEK-on-PK. There were no statistically significant differences in post-operative visual acuity although due to the nature of the study the groups were heterogenous. Surgeons should be prepared to offer any of these techniques depending on the merits of the clinical scenario and with careful attention to ocular or patient factors which may determine the choice of technique as well as the differences in failure rates compared to primary EK.

## Summary

### What was known before

- Prior studies have compared the results of endothelial transplant against repeat penetrating keratoplasty, or alternatively DMEK vs DSAEK in the management of failed penetrating keratoplasty.

### What this study adds

- This study is the first comparison of all three techniques for the management of endothelial failure of penetrating keratoplasty.

## Data Availability

Data are available upon written request to the corresponding author.
